# Association of postoperative navigated transcranial magnetic stimulation with accelerated motor recovery after tumor resection in the supplementary motor area

**DOI:** 10.1016/j.bas.2026.106167

**Published:** 2026-07-13

**Authors:** Boris Jeanquartier, Claire Descombes, Jonathan Wermelinger, Philippe Schucht, Anne Leyh, Nadja Zimmermann, Andreas Raabe, Kathleen Seidel

**Affiliations:** a Department of Neurosurgery, Inselspital, Bern University Hospital, Bern, Switzerland; b Department of Neurosurgery, Basel University Hospital, Basel, Switzerland; c Institute of Social and Preventive Medicine, University of Bern, Switzerland

**Keywords:** Transcranial magnetic stimulation, Supplementary motor area, Brain tumor, Motor eloquence, Brain plasticity, Rehabilitation

## Abstract

**Introduction:**

Following tumor resection in non-primary motor areas, patients may suffer from paresis. To distinguish between temporary and permanent deficits, our institution uses postoperative navigated transcranial magnetic stimulation (nTMS).

**Research question:**

We investigated the association of postoperative nTMS with motor recovery.

**Materials and methods:**

We retrospectively analysed the postoperative period in a cohort of 14 patients who underwent tumor resection in the supplementary motor area (SMA), suffered a postoperative paresis and underwent a single diagnostic nTMS session. The Medical Research Council Scale (MRCS) of the upper and lower limbs was added to obtain a MRCS-sum score (0-10). Patients were divided into early nTMS (<5 days after surgery) and late nTMS (≥5 days after surgery) groups, and changes in MRCS-sum scores were compared.

**Results:**

Following the nTMS session, MRCS-sum improvement was greater in the early nTMS group (2.1 ± 1.1; n = 9) than in the pre-stimulation interval in the late nTMS group (−0.3 ± 1.9; n = 5; Mann-Whitney U, p = 0.03). Improvements around the nTMS session were also larger in the early (2.1 ± 1.1) than in the late nTMS group (0.9 ± 0.9), although this difference did not reach statistical significance (p = 0.08). Linear regression identified the pre-nTMS MRCS-sum as the strongest predictor of post-nTMS improvement (p = 0.004).

**Discussion and conclusion:**

Following surgery for tumors in the SMA, our preliminary findings suggest an association between early nTMS (<5 days after surgery) and accelerated motor recovery from temporary paresis. Prospective trials are warranted.

## Highlights

• Early postoperative diagnostic nTMS shows an association with accelerated motor recovery after SMA tumor resection.
• Early diagnostic nTMS was followed by greater motor improvement than the natural recovery course.
• Exploratory findings warrant prospective controlled study of a facilitatory effect of a single diagnostic nTMS session.

## Abbreviations

IONM: Intraoperative Neurophysiological Monitoring
MEP: Motor Evoked Potential
MRCS: Medical Research Council Scale
nTMS: Navigated Transcranial Magnetic Stimulation
rTMS: Repetitive Transcranial Magnetic Stimulation
RMT: Resting Motor Threshold
SMA: Supplementary Motor Area

## 1 Introduction

Neuro-oncological surgery aims to balance between extent of tumor resection and preservation of neurological function (Chang et al., 2011; Smith et al., 2008; McGirt et al., 2009). To guide tumor resection near motor eloquent areas many techniques have been implemented in the operating room, including intraoperative neurophysiological monitoring and mapping (IONM) (De Witt Hamer et al., 2012; Sala et al., 2002; Seidel et al., 2022; Gerritsen et al., 2026). To estimate the distance to the corticospinal tract, the continuous dynamic mapping technique was introduced to provide real-time functional feedback aiming to reduce the risk of permanent deficits (Raabe et al., 2014; Seidel et al., 2013). Subsequent studies demonstrated that these methods allow safe and extended resections (Schucht et al., 2014; Moiyadi et al., 2018; Shiban et al., 2015; Roth et al., 2017; Seidel et al., 2025). In our centre, we apply a standardized IONM protocol with continuous monitoring of motor evoked potentials (MEPs) via a strip electrode placed on the precentral gyrus and subcortical continuous dynamic mapping via the suction probe (Seidel et al., 2025). However, particularly after resections involving non-primary motor areas such as the supplementary motor area (SMA) and the ventral premotor cortex (Mayka et al., 2006), postoperative motor deficits in the early days following surgery have been observed (Seidel et al., 2019, 2025).

One way of evaluating those early deficits is to use single-pulse navigated transcranial magnetic stimulation (nTMS). nTMS is a non-invasive tool used to assess the integrity of motor pathways (Picht et al., 2012; Krieg et al., 2013, 2017; Rossini et al., 2015). Recently, we demonstrated that postoperative nTMS predicts motor recovery after tumor resection with high accuracy (PPV = 90%, NPV = 100%) (Seidel et al., 2019). Other studies have demonstrated the same findings (Takakura et al., 2017; Eibl et al., 2025). The prognostic information has been proposed to support rehabilitation planning by identifying patients with favourable recovery potential (Seidel et al., 2019, 2020; Eibl et al., 2025). In parallel, studies using repetitive transcranial magnetic stimulation (rTMS), have provided first evidence for direct therapeutic effects on postsurgical paresis (Ille et al., 2021; Engelhardt et al., 2024; Rosenstock et al., 2025).

After confirming the predictive performance of nTMS regarding postoperative motor recovery, and motivated by our clinical observations, we aimed to examine the phenomenon of sudden postsurgical motor function improvement which appeared to be linked to a single, early postoperative diagnostic nTMS session. Therefore, we retrospectively analysed a patient cohort to explore whether single-pulse nTMS might play a role beyond prediction, that is, if it could potentially be used as a therapeutic impulse to facilitate motor recovery, similar to the therapeutic effects observed with rTMS.

While nTMS was applied solely in its established diagnostic role, the present analysis explores whether this single diagnostic session may have facilitated motor recovery in our cohort of patients which had undergone resection of tumors in the SMA.

## 2 Methods

### 2.1 Study design

The study was conducted in accordance with institutional guidelines and approved by the local ethics committee (KEK Bern, approval number: BASEC, 2024-00692). We retrospectively included patients who underwent resection of an intra-axial frontal tumor located in the SMA from September 2014 to December 2022. Inclusion criteria required that the patients developed a postoperative paresis and underwent a postoperative nTMS examination. Patients were excluded if the tumor affected the primary motor system, defined as involvement of the posterior precentral gyrus or corticospinal tract involvement on preoperative MRI.

We quantified motor strength by summing the Medical Research Council Scale (MRCS) grades of the contralateral upper and lower limb, yielding a total MRCS-sum from 0 to 10. This pragmatic approach ensured consistent scoring across the cohort which we considered feasible in the retrospective setting. Conceptually, this approach was analogous to additive motor scores established in neurorehabilitation, such as the SAFE score after stroke (Nijland et al., 2010) or the MRC sum score applied in Guillain-Barré syndrome (Kleyweg et al., 1991). Although the summation of ordinal MRC grades into a composite score involves an assumption of interval-level measurement that may not be fully justified, this pragmatic approach was considered reasonable in a retrospective setting and is analogous to established additive motor scores in neurorehabilitation (Nijland et al., 2010; Kleyweg et al., 1991). The resulting MRCS-sum was analysed throughout the peri- and postoperative course and in relation to the timing of the postoperative nTMS examination.

Additional variables collected included tumor histology, sex, age, nTMS stimulation side and significant changes in intraoperative MEPs. Significant MEP changes were classified in case of a sudden elevation of stimulation threshold of >4 mA during direct cortical MEP stimulation (via a strip electrode placed on the precentral gyrus) compared to baseline value or a decrement of >50% of MEP amplitude that could not be explained by technical (including accidental movement of the stimulation electrode) or anesthetic confounders (Seidel et al., 2025).

To assess whether nTMS timing influenced motor recovery, patients were divided into two groups: an early nTMS group (nTMS < day 5 post-op) and a late nTMS group (nTMS ≥ day 5). In both groups, we compared MRCS-sums before and after the nTMS session. Additionally, in the late nTMS group, initial motor recovery observed immediately after surgery and before stimulation was used as an approximation for the natural course of recovery.

The cut-off of five postoperative days was based mostly on clinical workflow considerations. As nTMS examinations are not performed on weekends, patients operated early in the week could typically undergo nTMS within the same working week. This situation was observed in 7 of 9 patients in the early group, while none of the late nTMS group patients were examined within the same working week their surgery took place. A cut-off at day 4 or earlier would have left the late nTMS group with insufficient pre-stimulation score measurements to approximate the natural course of recovery, while a cut-off at day 6 or later would have resulted in a markedly smaller and less balanced late nTMS group.

### 2.2 nTMS examination

nTMS was performed using a figure-of-eight coil with the Nexstim NBS System 4 (version 4.3) system before January 2021 and afterwards the Nexstim NBS System 5 (version 5.2). Overall, 11 of the included cases were performed with the old system, while three were performed with the new system. Since both of these systems use the same stimulator version and the same coils, they have the same maximum stimulator output and magnetic field.

Postoperative nTMS had been performed to determine whether an MEP could be elicited. If MEPs were observed in at least one muscle, the motor hotspot was identified. The resting motor threshold (RMT) was defined as the minimum stimulation intensity at which at least 5 out of 10 pulses elicited an MEP of amplitude ≥50 μV (Lenggenhager et al., 2025).

On average, approximately 60 stimulations were applied to the affected hemisphere and about 30 to the healthy hemisphere, adjusted according to patient tolerance, stimulation intensity (up to 100% if necessary), and MEP response (Seidel et al., 2019). Repetition rate of the single pulse stimulation (stimulation frequency) and stimulus counts were not systematically recorded in the clinical routine. However, based on our clinical routine the interstimulus interval between the single pulse stimulation was approximately 2-3 s, triggered manually by the TMS examiner.

### 2.3 Statistical analysis

Statistical analyses were performed using IBM SPSS Statistics (version 28) and R (version 4.1.2, R Core Team (2025)). Statistical significance was defined as *p* < 0.05.

Given the small sample size, categorical variables (nTMS group, sex, tumor location, stimulation side, tumor entity, intraoperative MEP threshold change) in the early nTMS and the late nTMS group were compared using Fisher's exact test, and continuous variables (age, score differences) and ordinal variables (motor scores, number of days) were compared using Mann-Whitney U tests.

**Fig. 1:**
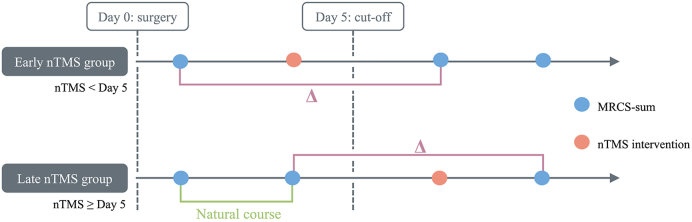
Visualization of the score measurement timepoints (blue dots) and nTMS intervention timepoints (orange dots) for the early and late nTMS groups. Δ represents the MRCS-sum difference around the nTMS session, calculated as the MRCS-sum at the later timepoint minus the MRCS-sum at the earlier timepoint. For the late nTMS group, the natural course interval additionally is used to approximate spontaneous recovery prior to nTMS. nTMS: navigated transcranial magnetic stimulation. For the definition of the MRCS-sum see methods section.

The time points used to compute the MRCS-sum differences are visualized in Fig. 1. For the early nTMS group, we calculated the difference in MRCS-sum before and after the nTMS session (difference in pink). In the late nTMS group, differences were computed over two different intervals: (1) the difference between two early time points before any nTMS session (mimicking natural recovery, in green), and (2) the difference in MRCS-sum before and after the late nTMS session (analogous to the early nTMS group, also in pink).

To account for potential confounders, we performed a linear regression using the MRCS-sum difference before (pre-nTMS MRCS-sum) and after (post-nTMS MRCS-sum) the nTMS examination as the response variable. As relevant covariates we included (1) the time point of nTMS examination (in days after surgery), (2) the intraoperative MEP threshold change (yes/no) and (3) the pre-nTMS MRCS-sum.

## 3 Results

**Fig. 2:**
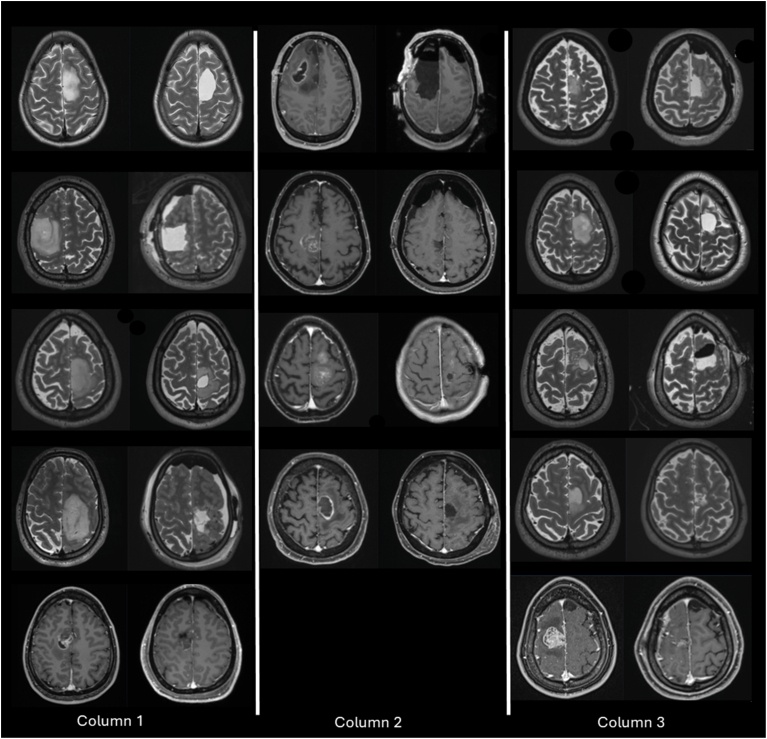
Early and late nTMS group patients with pre- and postoperative MRI-imaging. Columns 1 and 2 (left and middle column with respective pre- and postoperative MRI) represent the early nTMS group, and column 3 (right column with respective pre- and postoperative MRI) the late nTMS group. T1-weighted contrast-enhanced images are shown for high-grade gliomas and metastases, whereas T2-weighted images are shown for low-grade gliomas.

We included 14 patients into the study (Table 1): 9 in the early nTMS group and 5 in the late nTMS group. The median age was 45.1 years in the early nTMS group and 39.8 years in the late nTMS group (Mann-Whitney U, *p* = 0.61). Sex distribution was 4 female/5 male in the early nTMS group and 3 female/2 male in the late nTMS group (Fisher's exact test, *p* = 1.00). In a subset of 9 patients (Fig. 2), the lesion involved exclusively the SMA (5/9 in the early nTMS group vs. 4/5 in the late nTMS group), whereas in the remaining 5 patients the tumor and resection cavity extended beyond the SMA to include limited parts of the cingulate gyrus and/or the middle and superior frontal gyri (SMA plus: 4/9 vs. 1/5, respectively). These two categories were labelled as “SMA” and “SMA plus”. The distribution of these subgroups did not differ between early and late nTMS groups (p = 0.52). Subgroup analysis revealed no relevant differences in motor recovery around the time of nTMS between SMA and SMA plus patients. The hemisphere distribution was balanced in the early nTMS group (4 right, 5 left), while lesions were more often left-sided in the late nTMS group (4 left, 1 right) (Fisher's exact test, *p* = 0.58).

**Table 1:** Baseline characteristics.

Parameter	Early nTMS group (n = 9)	Late nTMS group (n = 5)	p-value
**Demographics**			
Median age (years)	45.1	39.8	0.61
Sex (female/male)	4/5	3/2	1.00
**Tumor Location**			
Supplementary Motor Area SMA (only/plus)	5/4	4/1	0.52
Hemisphere (left/right)	4/5	4/1	0.58
**Tumor Entity according to WHO Classification** (Louis et al., 2021)			
– Oligodendroglioma, IDH-mutant and 1p/19q-codeleted, CNS WHO grade 2	0	1	-
– Astrocytoma, IDH-mutant, CNS WHO grade 2	2	1	-
– Astrocytoma, IDH-mutant, CNS WHO grade 3	0	2	-
– Astrocytoma, IDH-mutant, CNS WHO grade 4	3	0	-
– Glioblastoma, IDH-wildtype, CNS WHO grade 4	3	0	-
– Metastasis	1	1	-
**Intraoperative Monitoring**			
Δ MEP threshold (yes/no)	4/5	0/5	0.22
**Motor Scores**			
Pre-nTMS MRCS-sum (0-10) (median)	3.5	8.0	0.01∗
**Timeline**			
Days from surgery to nTMS (median)	3.0	6.0	<0.01∗

The p-values correspond to a Mann-Whitney *U* test for continuous (age) and ordinal (MRCS-sum, days) variables and to Fisher's exact test for categorical variables. *A significance level of p < 0.05 was applied (indicated by an asterisk ∗)*.

Abbreviations: Δ MEP = intraoperative change in stimulation current required to elicit motor evoked potential (MEP); MRCS-sum = sum Medical Research Council Scale, for the definition see method section; SMA = supplementary motor area. The anatomical definitions of SMA and SMA plus is defined in the first paragraph of the result section.

Diagnoses according to the current WHO classification (Louis et al., 2021) included the following pathologies: In the early nTMS group there were two Astrocytomas, IDH-mutant, CNS WHO grade 2, three Astrocytoma, IDH-mutant, CNS WHO grade 4, three Glioblastoma, IDH-wildtype, CNS WHO grade 4, and one Metastasis. In the late nTMS group there was one Oligodendroglioma, IDH-mutant and 1p/19q-codeleted, CNS WHO grade 2, one Astrocytoma, IDH-mutant, CNS WHO grade 2, two Astrocytoma, IDH-mutant, CNS WHO grade 3 and one Metastasis.

A significant change in intraoperative MEP threshold or amplitude as described in the methods section during surgery was documented in 4 of 9 patients in the early nTMS group, whereas no such changes were observed in the late nTMS group (0 of 5 patients). (Fisher's exact test, *p* = 0.22; n = 14).

The pre-nTMS MRCS-sum (after surgery but before nTMS) was significantly lower in the early nTMS group (median 3.5) compared to the late nTMS group (median 8.0) (Mann-Whitney U, p = 0.01).

**Fig. 3:**
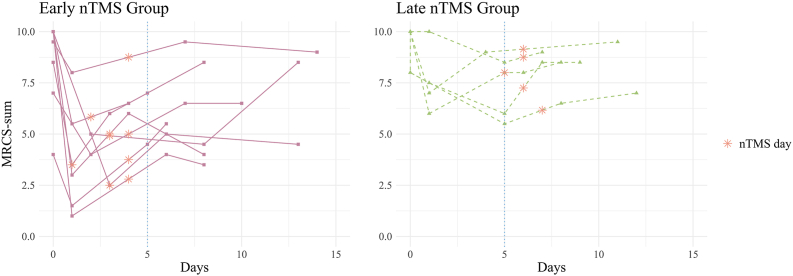
Timeline of MRCS-sum for all 9 patients in early nTMS group and all 5 patients in late nTMS group. Day 0 marks the day of surgery. Each orange asterisk represents the timing of the nTMS session**.** Solid pink lines (left side) indicate patients in the early nTMS group (nTMS < postoperative day 5), whereas dashed green lines (right side) represent patients in the late nTMS group. (nTMS ≥ postoperative day 5). Abbreviations: nTMS: navigated transcranial magnetic stimulation. MRCS-sum = sum Medical Research Council Scale, for the definition see method section.

For the improvement trajectories of the individual patients see Fig. 3 and Supplementary Fig. 1. Median MRCS-sum scores at the different time points are presented in Supplementary Fig. 2. The mean improvement in MRCS-sum after nTMS was significantly greater in the early nTMS group (2.1 ± 1.1) compared to the pre-stimulation recovery interval observed in the late nTMS group, which served as an approximation of the natural course in the absence of a true untreated control group (−0.3 ± 1.9; *p* = 0.03, Mann-Whitney *U* test). When comparing the score increments before and after nTMS within each group, the difference between the early (2.1 ± 1.1) and late nTMS group (0.9 ± 0.9) did not reach statistical significance (Mann-Whitney U, p = 0.08). As shown in Fig. 3, individual recovery trajectories varied considerably between patients within both groups, with patients in the early nTMS group presenting with significantly worse pre-nTMS MRCS-sum than patients in the late nTMS group (Table 1). The linear regression analysis (Supplementary Table 1) revealed that the pre-nTMS MRCS-sum was the only significant predictor of post-nTMS MRCS-sum improvement (*p* = 0.004), while neither nTMS timing nor intraoperative MEP changes reached significance. The R squared value was 0.63.

## 4 Discussion

In the described patient cohort with paresis after tumor resection in the SMA, our data suggests an accelerated motor recovery following a single diagnostic nTMS session. Although nTMS was applied solely for diagnostic purposes with single pulse stimulation, this effect has previously been reported in an individual case (Schmidt et al., 2013) but to our knowledge, it has never been studied beyond individual case reports. A possible explanation for this gap is that single pulse stimulation, given its low intensity compared with established repetitive protocols, may have been considered unlikely to produce a measurable clinical effect and therefore not pursued as a research question. Our exploratory observations raise the possibility that this assumption merits further prospective investigation.

### 4.1 Anatomical and functional considerations

The non-primary motor system plays a critical role in planning, initiating, executing, and correcting movements (Nachev et al., 2008). It includes the anterior cingulate cortex, posterior parietal cortex, dorsal and ventral premotor cortex and the SMA, all of which maintain di- or oligosynaptic connections to spinal α-motoneurons (Mayka et al., 2006; Nachev et al., 2008; Strick et al., 2021; Vesia and Crawford, 2012; Buneo and Andersen, 2006). Among these regions, the most distinctly anatomically defined region is the SMA. It is delineated caudally by the cingulate gyrus, extends approximately 2 cm anterior to the vertical plane through the anterior commissure, is bordered posteriorly by the precentral gyrus, and spans about 1.5 cm laterally from the midline (Mayka et al., 2006). Resection of tumors located in the SMA have been reported to cause an SMA syndrome, a mostly transient paresis that typically resolves within weeks to months, potentially through compensation by the contralateral SMA via transcallosal pathways (Pinson et al., 2022; Palmisciano et al., 2022; Di et al., 2024).

### 4.2 The role of TMS in facilitating/accelerating recovery and rehabilitation

Single-pulse nTMS is used in a diagnostic setting to evaluate primary motor cortex and corticospinal tract integrity, and thus to predict motor recovery potential for example after surgically induced postoperative paresis (Seidel et al., 2019, 2020; Eibl et al., 2025). Therapeutic applications of TMS typically involve high number of repetitive stimulations (rTMS) delivered over multiple sessions. These protocols include low-frequency stimulation (≤1 Hz) over the contralesional hemisphere to reduce transcallosal inhibition, and high-frequency stimulation (>5 Hz) over the ipsilesional primary motor cortex to enhance excitability (Rossini et al., 2015; Lefaucheur et al., 2020; Bai et al., 2022).

Low-frequency (inhibitory) contralesional TMS has shown beneficial effects in various populations, including healthy subjects, patients with stroke and in patients with postsurgical paresis (Ille et al., 2021; Engelhardt et al., 2024; Takeuchi et al., 2005; Engelhardt and Picht, 2020). A recent multicentre study protocol applied contralesional rTMS to reduce transcallosal inhibition for seven days starting on the first postoperative day, and the authors reported functional motor improvements. While the effect was particularly pronounced in subcortical ischemic injuries involving the corticospinal tract, improvements were also observed in patients with SMA syndrome (Rosenstock et al., 2025).

Multiple factors may account for the improvement in motor function observed in this study. Unlike conventional therapeutic protocols, our approach involved non-repetitive stimulation of mainly the lesioned hemisphere. In addition to stimulation of the affected hemisphere, limited stimulation was also applied to the contralesional non-affected hemisphere. This is part of our clinical routine and is conducted to elicit MEPs to provide a reference value of the non-affected side. While not intended as a therapeutic intervention, this stimulation may still have facilitated recovery via transcallosal inhibitory modulation. This stands in contrast to established rTMS rehabilitation protocols, which typically involve multiple daily sessions of repetitive stimulation over five to ten days (Ille et al., 2021; Engelhardt et al., 2024; Rosenstock et al., 2025; Lefaucheur et al., 2020).

The stimulation of the affected hemisphere may also have engaged recovery-relevant circuits. Possible neurophysiological mechanisms include transient disinhibition of ipsilesional cortical circuits via suppression of GABAergic interneurons (between M1 and SMA) (Engelhardt and Picht, 2020) and activation of inter-effector zones interconnected with the SMA and anterior cingulate cortex (Gordon et al., 2023), though these hypotheses remain speculative in the absence of direct neurophysiological evidence from the present cohort.

Beyond neurophysiology, a positive MEP response may increase reassurance and motivation, acting as biofeedback and indirectly facilitating recovery through enhanced participation in physiotherapy (Giggins et al., 2013).

### 4.3 Analysis results

The mean improvement in MRCS-sum after nTMS was significantly greater in the early nTMS group compared to the late nTMS group prior to stimulation. The regression analysis showed that the pre-nTMS MRCS-sum was the only significant predictor of post-nTMS MRCS-sum improvement (p = 0.004), while the timing of nTMS examination and intraoperative MEP changes did not significantly contribute. This suggests that the observed between-group differences may be partially attributable to differences in baseline motor function rather than to the timing of stimulation per se, a possibility addressed further in the Limitations section. The model explained a substantial proportion of the variance (R^2^ = 0.632), suggesting that the predictor variables were suitably selected. Despite the constraints discussed below, the consistent observation of a rapid recovery after nTMS in several individual patients, together with recent large-scale studies reporting postoperative rTMS-induced improvements (Ille et al., 2021; Engelhardt et al., 2024; Rosenstock et al., 2025), suggests that this question warrants further investigation in adequately powered prospective studies.

### 4.4 Our findings in the context of existing evidence

While the therapeutic potential of rTMS is well established, evidence for direct therapeutic effects of single-pulse nTMS remains limited. Randomized controlled trials have demonstrated that navigated rTMS can significantly improve recovery from postsurgical paresis in glioma patients (Ille et al., 2021; Engelhardt et al., 2024), and these findings are supported by large-scale clinical data (Rosenstock et al., 2025). Similar approaches have been established in stroke rehabilitation, where rTMS has been shown to promote recovery by modulating cortical excitability and interhemispheric balance (Takeuchi et al., 2005). Although therapeutic effects of single-pulse nTMS on motor recovery have not been studied in more than a single patient, one case report described unexpected functional gains after an intensive nTMS session (Schmidt et al., 2013). Together with the association between postoperative nTMS and early motor recovery observed in our cohort, these findings suggest that the effects of single pulse nTMS may extend beyond its established diagnostic and prognostic roles, and may serve as a therapeutic impulse in the early course after tumor resection in non-primary motor areas, especially the SMA.

Prospective, controlled studies are needed to control for confounding, and to determine whether this effect is reproducible and how stimulation parameters and paradigms could be optimized to exploit this response clinically. Further, the focus on the lesional hemisphere during stimulation warrants further investigation. This is in line with an emerging shift in stroke rehabilitation, where excitatory stimulation protocols directed at the ipsilesional hemisphere have shown promising effects on upper limb motor recovery, complementing the more established contralesional inhibitory paradigms (Tang et al., 2022).

## 5 Limitations

This study has several limitations. Its retrospective nature means that motor function was documented using simplified clinical scales instead of standardized instruments. The small sample size (n = 14) severely limits statistical power and generalizability, and the regression model is therefore likely underpowered and potentially unstable, with its results warranting considerable caution. The choice of postoperative day 5 as the cut-off, while based on clinical workflow considerations, remains arbitrary. The resulting grouping was therefore largely driven by the weekday of surgery and the severity of the postoperative deficit, and the choice of day 5 as the threshold also reflected the need to maintain a practicable group size on both sides of the cut-off and to be able to compare the natural course of recovery in the late nTMS group with the early nTMS group, rather than representing a pre-specified or validated clinical criterion. Further, the pre-stimulation recovery interval observed in the late nTMS group served as an approximation of the natural course of recovery, which does not constitute a true control group without stimulation and therefore precludes conclusions about causality. Similarly, the absence of sham stimulation means that we cannot exclude a placebo effect in the recovery evolution of our cohort. Additionally, patients with more pronounced deficits were more likely to receive early nTMS, as the prognostic information was needed sooner to guide rehabilitation planning and patient counselling. This is reflected in the substantially lower pre-nTMS MRCS-sum in the early nTMS group compared to the late nTMS group, introducing a systematic difference in baseline prognostic factors between groups and a potential confounding by indication that cannot be fully controlled for in the retrospective setting. This confounding by indication in the early group may partially explain the larger observed improvement, while the relatively preserved motor function at baseline in the late group may have reduced the range of potential measurable response, creating a ceiling effect. These opposing sources of bias cannot be excluded in this retrospective design, and together with the considerable interindividual variability in recovery trajectories, they underscore the limited generalizability of group-level findings in this small cohort and suggest that patient-specific factors beyond those captured in the regression model may influence recovery after nTMS. Furthermore, the absence of systematically recorded stimulation parameters such as pulse counts and repetition rate of single pulses during manual stimulation represents an important limitation of this study. As these factors may be relevant to any facilitatory effect on motor recovery, their unavailability limits mechanistic interpretation of the present findings. Finally, heterogeneity in follow-up intervals complicates temporal interpretation of motor recovery trajectories. However, this study might provide a basis for a refined prospective protocol.

## 6 Conclusion

The present retrospective analysis suggests a potential association between early postoperative nTMS and accelerated motor recovery following tumor resection in the SMA. Given the absence of a proper control group and the very limited sample size, causal conclusions cannot be drawn. Prospective, controlled studies are needed to determine if this association is reproducible and if single pulse nTMS on the lesional hemisphere may have a role beyond its established diagnostic function.

## Author contributions

The study was conceptualized and designed by Boris Jeanquartier and Kathleen Seidel. Data collection and clinical chart review were performed by Boris Jeanquartier. Statistical analyses were conducted by Jonathan Wermelinger, Claire Descombes, and Boris Jeanquartier. Figure preparation was carried out by Claire Descombes and Boris Jeanquartier. Intraoperative neuromonitoring (IONM) data were provided and analysed by Anne Leyh. The manuscript was drafted by Boris Jeanquartier and critically revised by Kathleen Seidel, Jonathan Wermelinger, Claire Descombes, Philippe Schucht, Nadja Zimmermann and Andreas Raabe. All authors have read and approved the final version of the manuscript for submission.

## Data availability

The anonymized datasets generated and analysed during the current study are available from the corresponding author upon reasonable request and with appropriate ethics committee approval.

## Funding

No external funding was received for this study.

## Declaration of competing interest

The authors declare that they have no known competing financial interests or personal relationships that could have appeared to influence the work reported in this paper.
