# Supplementary material for: Association of postoperative navigated transcranial magnetic stimulation with accelerated motor recovery after tumor resection in the supplementary motor area
Source: Brain Spine. 2026 Jul 13;6:106167. doi: 10.1016/j.bas.2026.106167 (PMC13400417; doi:10.1016/j.bas.2026.106167)
Supplement: Multimedia component 1 [file mmc1.doc]

# Supplements


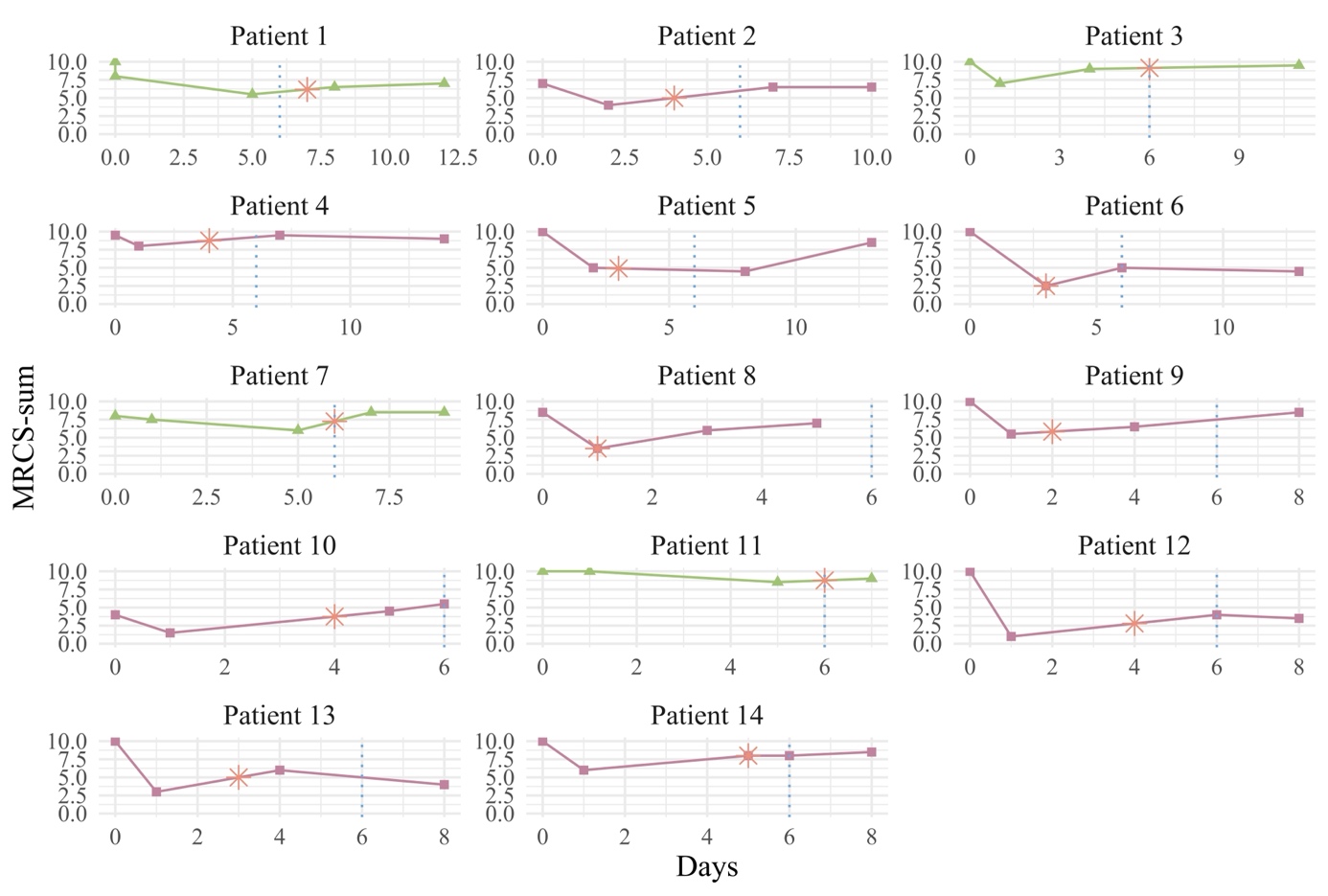


*Supplementary Figure 1. Individual MRCS-sum trajectories for each patient over time. The x-axis indicates time in days, and the y-axis represents the MRCS-sum score.* *Day 0 marks the day of surgery. Each orange asterisk represents the timing of the nTMS session. Solid pink lines indicate patients in the early nTMS group (nTMS < postoperative day 5), whereas dashed green lines represent patients in the late nTMS group. (nTMS ≥ postoperative day 5).*
*Abbreviation: MRCS-sum = sum Medical Research Council Scale, for the definition see method section*


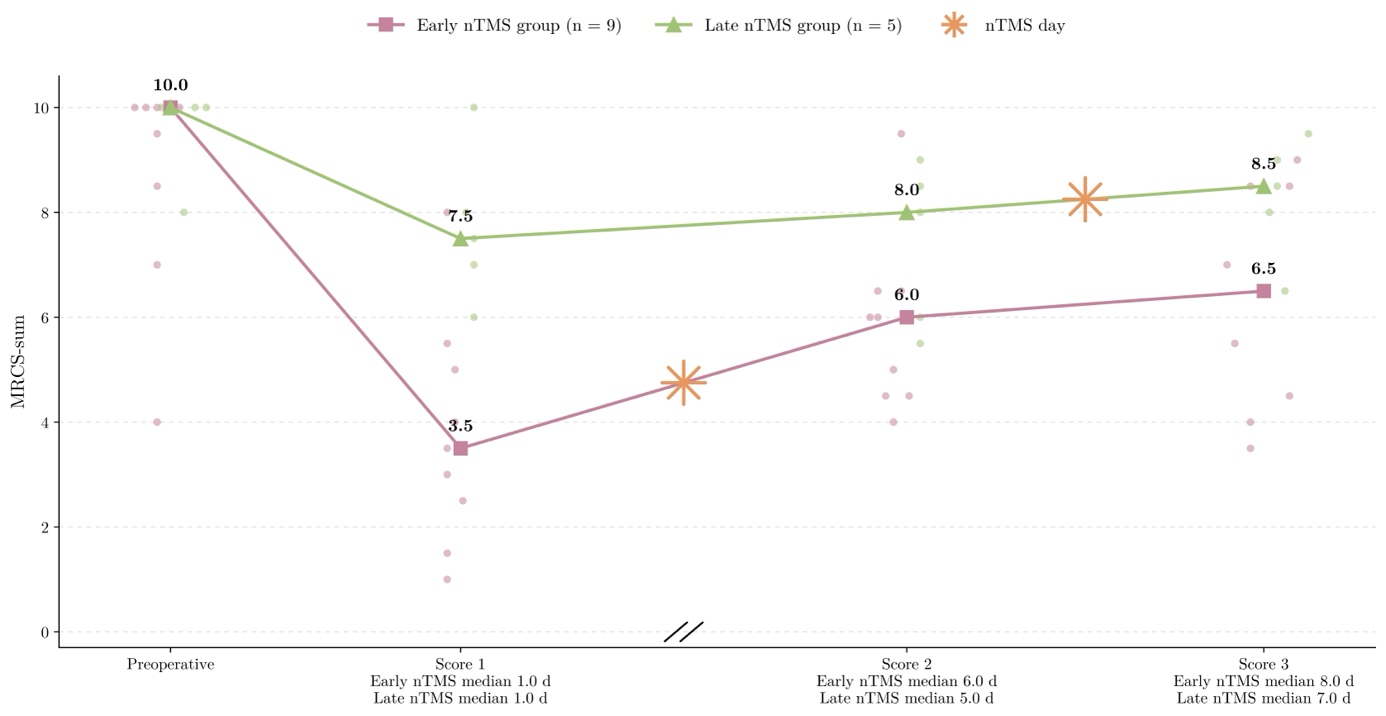


*Supplementary Figure 2. Median MRCS-sum trajectories at each assessment timepoint for the early nTMS group (squares, n = 9) and late nTMS group (triangles, n = 5). Labeled values represent group medians. Individual patient MRCS-sums are shown as colour-coded dots. The diagonal break marks (//) on the x-axis indicate the omitted time interval between the first postoperative assessment and the subsequent timepoints. Asterisks indicate the timing of the nTMS session (between MRCS-sum 1 and MRCS-sum 2 for the early nTMS group; between MRCS-sum 2 and MRCS-sum 3 for the late nTMS group). nTMS = navigated transcranial magnetic stimulation. MRCS-sum = sum Medical Research Council Scale; for the definition see methods section.*

**Supplementary Table 1: Linear regression model**

| Model | |  | | t-value | p-value |
| --- | --- | --- | --- | --- | --- |
|  |  | Unst. Coeff. B | Std. Error |  |  |
| 1 | (Constant) | 3.853 | 0.808 | 4.767 | **0.001*** |
|  | Time point of nTMS | 0.103 | 0.159 | 0.649 | 0.531 |
|  | MEP Changes | -0.850 | 0.631 | -1.346 | 0.208 |
|  | Pre-nTMS MRCS-sum | -0.472 | 0.127 | -3.727 | **0.004*** |

*Estimated parameters of a multiple linear regression model (n = 14) with the difference in MRCS-sum between post- and pre-nTMS measurements as the response variable. Covariates included time point of nTMS, MEP changes, and pre-nTMS MRCS-sum. Unstandardized regression coefficients (B) with corresponding standard errors, t-values, and two-sided p-values are shown. A significance level of p < 0.05 was applied (indicated by an asterisk *). R² = 0.632. No correction for multiple testing was performed.*

*Abbreviations: nTMS, navigated transcranial magnetic stimulation; MEP, motor evoked potential; MRCS-sum = sum Medical Research Council Scale, for the definition see method section*
